# Joint distribution approaches to simultaneously quantifying benefit and risk

**DOI:** 10.1186/1471-2288-6-48

**Published:** 2006-10-12

**Authors:** Michele L Shaffer, Kristi L Watterberg

**Affiliations:** 1 Departments of Health Evaluation Sciences and Pediatrics, Penn State College of Medicine, Hershey, PA, USA; 2 Department of Pediatrics/Neonatology, University of New Mexico, Albuquerque, NM, USA

## Abstract

**Background:**

The benefit-risk ratio has been proposed to measure the tradeoff between benefits and risks of two therapies for a single binary measure of efficacy and a single adverse event. The ratio is calculated from the difference in risk and difference in benefit between therapies. Small sample sizes or expected differences in benefit or risk can lead to no solution or problematic solutions for confidence intervals.

**Methods:**

Alternatively, using the joint distribution of benefit and risk, confidence regions for the differences in risk and benefit can be constructed in the benefit-risk plane. The information in the joint distribution can be summarized by choosing regions of interest in this plane. Using Bayesian methodology provides a very flexible framework for summarizing information in the joint distribution.

**Results:**

Data from a National Institute of Child Health & Human Development trial of hydrocortisone illustrate the construction of confidence regions and regions of interest in the benefit-risk plane, where benefit is survival without supplemental oxygen at 36 weeks postmenstrual age, and risk is gastrointestinal perforation. For the subgroup of infants exposed to chorioamnionitis the confidence interval based on the benefit-risk ratio is wide (Benefit-risk ratio: 1.52; 90% confidence interval: 0.23 to 5.25). Choosing regions of appreciable risk and acceptable risk in the benefit-risk plane confirms the uncertainty seen in the wide confidence interval for the benefit-risk ratio – there is a greater than 50% chance of falling into the region of acceptable risk – while visually allowing the uncertainty in risk and benefit to be shown separately. Applying Bayesian methodology, an incremental net health benefit analysis shows there is a 72% chance of having a positive incremental net benefit if hydrocortisone is used in place of placebo if one is willing to incur at most one gastrointestinal perforation for each additional infant that survives without supplemental oxygen.

**Conclusion:**

If the benefit-risk ratio is presented, the joint distribution of benefit and risk also should be shown. These regions avoid the ambiguity associated with collapsing benefit and risk to a single dimension. Bayesian methods allow even greater flexibility in simultaneously quantifying benefit and risk.

## Background

When comparing the effects of a new therapy with an existing therapy, it is not uncommon for the new therapy to show increased risks along with increased benefits. We consider the case of a single binary measure of efficacy and a single binary measure of risk or adverse event (absent/present, ever/never) and address the questions:

1. How do you appropriately measure the tradeoff between the benefit and risk of two therapies?

2. When should you conclude the increased benefit of a new therapy outweighs the potential increased risk?

Rather than focusing on hypothesis testing and controlling the type I error rate, our interest is in jointly quantifying benefit and risk.

### The benefit-risk ratio

One method that has been suggested for measuring the tradeoff between a binary measure of benefit and a binary measure of risk is the benefit-risk ratio [1]. The benefit-risk ratio is the ratio of the difference in benefit to difference in risk, or equivalently, the ratio of Number Needed to Harm (NNH) to Number Needed to Treat (NNT):

R=pE−pCqE−qC=NNHNNT
 MathType@MTEF@5@5@+=feaafiart1ev1aaatCvAUfKttLearuWrP9MDH5MBPbIqV92AaeXatLxBI9gBaebbnrfifHhDYfgasaacH8akY=wiFfYdH8Gipec8Eeeu0xXdbba9frFj0=OqFfea0dXdd9vqai=hGuQ8kuc9pgc9s8qqaq=dirpe0xb9q8qiLsFr0=vr0=vr0dc8meaabaqaciaacaGaaeqabaqabeGadaaakeaacqWGsbGucqGH9aqpdaWcaaqaaiabdchaWnaaBaaaleaacqWGfbqraeqaaOGaeyOeI0IaemiCaa3aaSbaaSqaaiabdoeadbqabaaakeaacqWGXbqCdaWgaaWcbaGaemyraueabeaakiabgkHiTiabdghaXnaaBaaaleaacqWGdbWqaeqaaaaakiabg2da9maalaaabaGaemOta4KaemOta4KaemisaGeabaGaemOta4KaemOta4Kaemivaqfaaaaa@4381@

where *p*_*E *_and *p*_*c *_are the probabilities of benefit in the experimental treatment and control arms, respectively, and *q*_*E *_and *q*_*c *_are the probabilities of risk in the experimental treatment and control arms, respectively.

**Figure 1:**
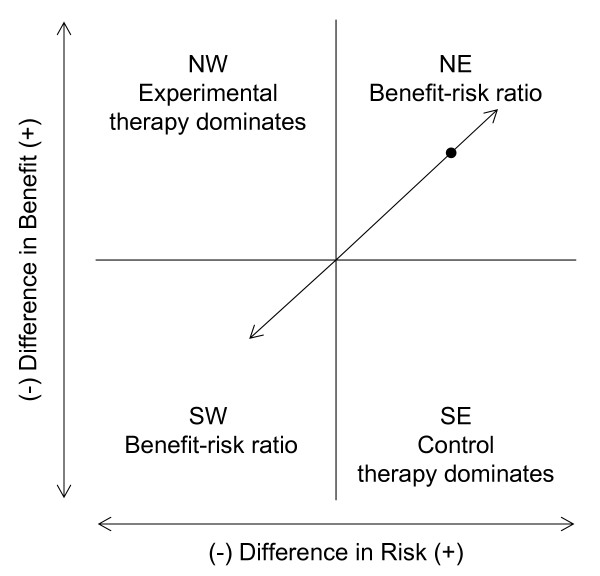
**The benefit-risk ratio in the benefit-risk plane**. The benefit-risk ratio is the slope of the line which passes through the origin and the point defined by the observed difference in risk and observed difference in benefit.

The benefit-risk ratio can be interpreted as the increase in the number of expected patients who will benefit for each additional adverse event that is incurred from using the experimental treatment rather than the control. The ratio also can be viewed in the benefit-risk plane as the slope of the line that passes through the origin and point defined by the observed difference in risk and difference in benefit as shown in Figure 1. The benefit-risk ratio is similar to the incremental cost-effectiveness ratio (ICER), which measures the tradeoff between the cost and effectiveness of two therapies. The ICER is defined as the ratio of the mean treatment difference in cost to the mean treatment difference in effectiveness for two therapies:

ICER=γE−γCεE−εC
 MathType@MTEF@5@5@+=feaafiart1ev1aaatCvAUfKttLearuWrP9MDH5MBPbIqV92AaeXatLxBI9gBaebbnrfifHhDYfgasaacH8akY=wiFfYdH8Gipec8Eeeu0xXdbba9frFj0=OqFfea0dXdd9vqai=hGuQ8kuc9pgc9s8qqaq=dirpe0xb9q8qiLsFr0=vr0=vr0dc8meaabaqaciaacaGaaeqabaqabeGadaaakeaacqWGjbqscqWGdbWqcqWGfbqrcqWGsbGucqGH9aqpdaWcaaqaaGGaciab=n7aNnaaBaaaleaacqWGfbqraeqaaOGaeyOeI0Iae83SdC2aaSbaaSqaaiabdoeadbqabaaakeaacqWF1oqzdaWgaaWcbaGaemyraueabeaakiabgkHiTiab=v7aLnaaBaaaleaacqWGdbWqaeqaaaaaaaa@3FAC@

where *γ*_*E *_and *γ*_*C *_are average costs of the experimental and control conditions, respectively, and *ε*_*E*_and *ε*_*C *_are average effectiveness measures of the experimental and control conditions, respectively. One can similarly view the ICER in the cost-effectiveness plane. Distributional assumptions may differ for the benefit-risk ratio and cost-effectiveness ratio with cost generally considered a continuous measure. And while effectiveness appears in the denominator of the ICER, benefit is in the numerator of the benefit-risk ratio. Furthermore, although the current discussion focuses on a single binary measure of risk, consolidating multiple risks into a single measure may be more problematic than combining costs.

There is some ambiguity in reducing the difference in benefit and difference in risk to a single measure. As differing magnitudes of benefit and risk can result in the same ratio, control therapy could show more benefit and more risk and yield the same ratio as a new therapy which shows more benefit and more risk. Note in Figure 1 that any observed difference in benefit and observed difference in risk that falls on the line shown through the origin will produce the same benefit-risk ratio. For example, suppose the difference in benefit favors the new therapy over control and is 0.30, but the new therapy also increases the adverse event rate by 0.20; the resulting benefit-risk ratio is 1.5. However, if the difference in benefit favors control over the new therapy and is -0.30, but the new therapy reduces the adverse event rate by 0.20, then the resulting benefit risk ratio also is 1.5. When deciding whether the new therapy is acceptable, it is unlikely that these two scenarios would be considered equivalent. In the first scenario we are weighing increased benefit against increased risk, while in the latter we are weighing decreased benefit against decreased risk. Heitjan et al. highlighted similar complications for estimation of the ICER [2].

Confidence intervals can be constructed for the benefit-risk ratio using methods similar to those used to compute confidence intervals for cost-effectiveness ratios [3-5]. Assuming bivariate normality, Willan et al. showed that Fieller's theorem can be used to compute confidence intervals where the variance of the bivariate normal distribution is given by

V([q^E−q^C,p^E−p^C]′)=[qE(1−qE)nE+qC(1−qC)nCbE−pEqEnE+bC−pCqCnCbE−pEqEnE+bC−pCqCnCpE(1−pE)nE+pC(1−pC)nC]
 MathType@MTEF@5@5@+=feaafiart1ev1aaatCvAUfKttLearuWrP9MDH5MBPbIqV92AaeXatLxBI9gBaebbnrfifHhDYfgasaacH8akY=wiFfYdH8Gipec8Eeeu0xXdbba9frFj0=OqFfea0dXdd9vqai=hGuQ8kuc9pgc9s8qqaq=dirpe0xb9q8qiLsFr0=vr0=vr0dc8meaabaqaciaacaGaaeqabaqabeGadaaakeaacqWGwbGvcqGGOaakcqGGBbWwcuWGXbqCgaqcamaaBaaaleaacqWGfbqraeqaaOGaeyOeI0IafmyCaeNbaKaadaWgaaWcbaGaem4qameabeaakiabcYcaSiqbdchaWzaajaWaaSbaaSqaaiabdweafbqabaGccqGHsislcuWGWbaCgaqcamaaBaaaleaacqWGdbWqaeqaaOGafiyxa0LbauaacqGGPaqkcqGH9aqpdaWadaqaauaabeqaceaaaeaadaWcaaqaaiabdghaXnaaBaaaleaacqWGfbqraeqaaOGaeiikaGIaeGymaeJaeyOeI0IaemyCae3aaSbaaSqaaiabdweafbqabaGccqGGPaqkaeaacqWGUbGBdaWgaaWcbaGaemyraueabeaaaaGccqGHRaWkdaWcaaqaaiabdghaXnaaBaaaleaacqWGdbWqaeqaaOGaeiikaGIaeGymaeJaeyOeI0IaemyCae3aaSbaaSqaaiabdoeadbqabaGccqGGPaqkaeaacqWGUbGBdaWgaaWcbaGaem4qameabeaaaaGcdaWcaaqaaiabdkgaInaaBaaaleaacqWGfbqraeqaaOGaeyOeI0IaemiCaa3aaSbaaSqaaiabdweafbqabaGccqWGXbqCdaWgaaWcbaGaemyraueabeaaaOqaaiabd6gaUnaaBaaaleaacqWGfbqraeqaaaaakiabgUcaRmaalaaabaGaemOyai2aaSbaaSqaaiabdoeadbqabaGccqGHsislcqWGWbaCdaWgaaWcbaGaem4qameabeaakiabdghaXnaaBaaaleaacqWGdbWqaeqaaaGcbaGaemOBa42aaSbaaSqaaiabdoeadbqabaaaaaGcbaWaaSaaaeaacqWGIbGydaWgaaWcbaGaemyraueabeaakiabgkHiTiabdchaWnaaBaaaleaacqWGfbqraeqaaOGaemyCae3aaSbaaSqaaiabdweafbqabaaakeaacqWGUbGBdaWgaaWcbaGaemyraueabeaaaaGccqGHRaWkdaWcaaqaaiabdkgaInaaBaaaleaacqWGdbWqaeqaaOGaeyOeI0IaemiCaa3aaSbaaSqaaiabdoeadbqabaGccqWGXbqCdaWgaaWcbaGaem4qameabeaaaOqaaiabd6gaUnaaBaaaleaacqWGdbWqaeqaaaaakmaalaaabaGaemiCaa3aaSbaaSqaaiabdweafbqabaGccqGGOaakcqaIXaqmcqGHsislcqWGWbaCdaWgaaWcbaGaemyraueabeaakiabcMcaPaqaaiabd6gaUnaaBaaaleaacqWGfbqraeqaaaaakiabgUcaRmaalaaabaGaemiCaa3aaSbaaSqaaiabdoeadbqabaGccqGGOaakcqaIXaqmcqGHsislcqWGWbaCdaWgaaWcbaGaem4qameabeaakiabcMcaPaqaaiabd6gaUnaaBaaaleaacqWGdbWqaeqaaaaaaaaakiaawUfacaGLDbaaaaa@A39A@

where "hats" indicate the observed values of population parameters and *b*_*E *_and *b*_*C *_are the probabilities of simultaneous benefit and risk in the same subject for the experimental treatment and control arms, respectively [1]. The variance is estimated (V^
 MathType@MTEF@5@5@+=feaafiart1ev1aaatCvAUfKttLearuWrP9MDH5MBPbIqV92AaeXatLxBI9gBaebbnrfifHhDYfgasaacH8akY=wiFfYdH8Gipec8Eeeu0xXdbba9frFj0=OqFfea0dXdd9vqai=hGuQ8kuc9pgc9s8qqaq=dirpe0xb9q8qiLsFr0=vr0=vr0dc8meaabaqaciaacaGaaeqabaqabeGadaaakeaacuWGwbGvgaqcaaaa@2DF1@) by replacing the population parameters with the observed values. Calculation of the confidence limits by Fieller's theorem involves matrix manipulation which can be done in several packages including PROC IML in SAS (SAS Institute, Inc., Cary, NC), Mathematica (Wolfram Research, Inc., Champaign, IL), S-PLUS (Insightful Corporation, Seattle, WA), or the free software R [6]. Alternatively, the bootstrap can be used to construct confidence intervals using the percentile method [7].

**Figure 2:**
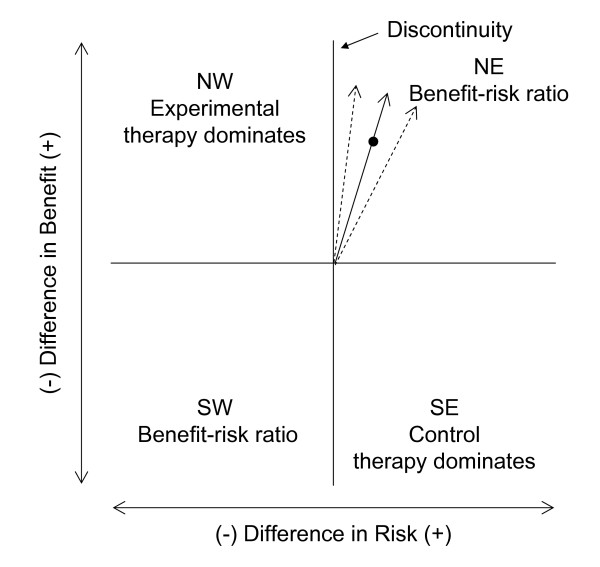
**Confidence limits in the benefit-risk plane**. The confidence limits of the benefit-risk ratio can be represented as slopes of lines (dotted) which pass through the origin. A discontinuity exists when the difference in risk is 0.

Difficulties can arise in using either Fieller's theorem or the bootstrap methods to construct confidence intervals [1,8,4,2,9]. Intractable or problematic solutions can result using Fieller's theorem because of small sample sizes and/or small expected differences in benefit and/or risk. As shown in Figure 2, the confidence limits of the benefit-risk ratio also can be represented as slopes of lines in the benefit-risk plane, and there is a discontinuity in the distribution of the benefit-risk ratio when the difference in risk is 0. For the bootstrap method, it may be unclear how to order estimates from the bootstrap samples when they fall in multiple quadrants. Heitjan et al. proposed reordering the bootstrap samples for the ICER (modified percentile bootstrap), taking into account the quadrant in which the ratio falls [4]. A more complete solution by Heitjan et al. uses Bayesian methodology and treats the ICER as a two-dimensional parameter composed of the ICER value and the quadrant in which the effectiveness difference and cost difference fall [2]. This methodology has been extended to handle censored effectiveness data [9].

### Other simultaneous measures of benefit and risk

Other measures have been suggested to summarize differences in benefit and risk. An early example is the work by Tallarida et al. on a severity scale developed through physician interviews which synthesizes information on disease severity and adverse drug reactions so that these considerations can be quantitatively incorporated into a benefit-risk analysis [10]. Chuang-Stein et al. presented three ratio measures that require assigning weights to categories of the form: (1) benefit without adverse event, (2) benefit with adverse event, (3) no benefit and no adverse event, (4) no benefit with adverse event, and (5) unacceptable adverse event leading to withdrawal [11]. While these ratios are more general than the benefit-risk ratio, specifying weights that reflect the relative importance of the categories may be difficult. Later work by Chuang-Stein discounts benefit by risk using consolidated safety data [12,13]. As noted by Holden, these approaches do not clearly delineate benefit and risk which makes their interpretation more complicated than the traditional benefit-risk ratio [14].

## Methods

### Confidence regions

Rather than collapsing the difference in benefit and difference in risk into a single dimension, the joint density of benefit and risk can be represented in the benefit-risk plane. Similar methods have been proposed for cost-effectiveness analyses [15,16]. Confidence regions can be constructed either under the bivariate normal assumption or using the bootstrap and nonparametric density estimation. Assuming bivariate normality, the confidence region is an ellipse. To construct a nonparametric confidence region, we draw repeated (bootstrap) samples with replacement and compute a benefit difference and risk difference for each of the samples. Next we obtain a two-dimensional kernel density estimate using the set of bootstrap estimates and find a contour of the kernel density estimate that includes (1 - *α*) × 100% of the bootstrap estimates [17]. Two-dimensional kernel density estimation methods are available for S-PLUS or R.

In addition to plotting the confidence region in the benefit-risk plan, we also can partition the benefit-risk plane into chosen regions of interest, e.g.,

**1**. Appreciable risk

**2**. No appreciable benefit

**3**. No conclusion ("gray region")

**4**. Experimental therapy superior

and look at the proportion of bootstrap estimates that fall into each region. These regions may be easier to specify for the clinician than the weights needed for the weighted benefit-risk ratios proposed by Chuang-Stein et al. [11].

### Bayesian methods

As an alternative to the confidence region approach, using asymptotic theory, Bayesian inference can be based on the posterior distribution of the difference in benefit and difference in risk, assuming that the prior distribution is locally uniform (or continuous and nonzero) near the true difference in risk and difference in benefit [18]. Using the posterior distribution,

p([qE−qC,pE−pC]′|[q^E−q^C,p^E−p^C]′)≈N([q^E−q^C,p^E−p^C]′,V^)
 MathType@MTEF@5@5@+=feaafiart1ev1aaatCvAUfKttLearuWrP9MDH5MBPbIqV92AaeXatLxBI9gBaebbnrfifHhDYfgasaacH8akY=wiFfYdH8Gipec8Eeeu0xXdbba9frFj0=OqFfea0dXdd9vqai=hGuQ8kuc9pgc9s8qqaq=dirpe0xb9q8qiLsFr0=vr0=vr0dc8meaabaqaciaacaGaaeqabaqabeGadaaakeaacqWGWbaCcqGGOaakcqGGBbWwcqWGXbqCdaWgaaWcbaGaemyraueabeaakiabgkHiTiabdghaXnaaBaaaleaacqWGdbWqaeqaaOGaeiilaWIaemiCaa3aaSbaaSqaaiabdweafbqabaGccqGHsislcqWGWbaCdaWgaaWcbaGaem4qameabeaakiqbc2faDzaafaGaeiiFaWNaei4waSLafmyCaeNbaKaadaWgaaWcbaGaemyraueabeaakiabgkHiTiqbdghaXzaajaWaaSbaaSqaaiabdoeadbqabaGccqGGSaalcuWGWbaCgaqcamaaBaaaleaacqWGfbqraeqaaOGaeyOeI0IafmiCaaNbaKaadaWgaaWcbaGaem4qameabeaakiqbc2faDzaafaGaeiykaKIaeyisISRaemOta4KaeiikaGIaei4waSLafmyCaeNbaKaadaWgaaWcbaGaemyraueabeaakiabgkHiTiqbdghaXzaajaWaaSbaaSqaaiabdoeadbqabaGccqGGSaalcuWGWbaCgaqcamaaBaaaleaacqWGfbqraeqaaOGaeyOeI0IafmiCaaNbaKaadaWgaaWcbaGaem4qameabeaakiqbc2faDzaafaGaeiilaWIafmOvayLbaKaacqGGPaqkaaa@6892@

the posterior probability of falling into the chosen regions can be computed [19]. The integration required can be carried out using the numerical integration function *N Integrate *in Mathematica or similar software. The probability interpretation of the Bayesian analysis is more straightforward than the confidence interpretation associated with the bootstrapping approach.

Decision analysis also can be conducted under the Bayesian framework using linear combinations of the form

*f*(*A*, *B*) = *A*(*p*_*E *_- *p*_*C*_) - *B*(*q*_*E *_- *q*_*C*_)

Point estimates and probability intervals for these linear combinations can be computed by taking a large number of draws from the posterior distribution and computing f(A, B) for each draw. The median of the draws can be used as a point estimate of f(A, B), and the 100*α*/2 and 100(1 - *α*/2) centiles of these draws form a 100(1 - *α*)% interval estimate.

These linear combinations also can be used to conduct benefit-risk analyses analogous to the incremental net health benefit (*INHB*)approach used in cost-effectiveness analyses [20,21]. In the cost-effectiveness setting, the *INHB *of an experimental treatment compared to a control is defined as

*INHB*(*λ*) = (*ε*_*E *_- *ε*_*C*_) - (*γ*_*E *_- *γ*_*C*_)/*λ*

where *λ *can be thought of as the maximum society is willing to pay for an incremental gain in health [20]. One obvious advantage of this approach is that INHB is measured in units of effectiveness so the quadrant ambiguity of the cost-effectiveness approach is no longer an issue.

Analogously, in the benefit-risk setting, we'll define an incremental health benefit of the experimental therapy compared to the control as

*INHB*_*BR*_(*δ*) = (*p*_*E *_- *p*_*C*_) - (*q*_*E *_- *q*_*C*_)/*δ*

where *δ *can be thought of as the maximum number of adverse events one is willing to incur for each subject that benefits. Alternatively, and perhaps more meaningfully, one can interpret 1/*δ *as the minimum number of subjects who should benefit for each additional adverse event. Integration over the posterior distribution of the risk difference and benefit difference can be used to compute *Pr*[*INHB*_*BR*_(*δ*) > 0] for a particular *δ *value or one can look at a plot of *Pr*[*INHB*_*BR*_(*δ*) > 0] over a range of *δ *values.

Although we have used large sample theory to assume the posterior distribution of the difference in risk and difference in benefit is bivariate normal, this assumption is not necessary for these Bayesian methods. As long as it is possible to simulate draws from the posterior distribution, these point estimates and probability intervals can be calculated under other distributional assumptions. Simulation approximations to the integration required to compute the posterior probabilities, *Pr*[*INHB*_*BR*_(*δ*) > 0], are obtained by computing the percentage of simulation draws for which *INHB*_*BR*_(*δ*) exceeds 0. Similar simulation approximations to integration can be used to compute posterior probabilities of falling into chosen regions of interest in the benefit-risk plane.

## Results and discussion

The PROPHET study is a multicenter, randomized clinical trial comparing placebo (n = 180) to low-dose hydrocortisone therapy (n = 180) in the first two weeks of life in extremely low birth weight babies (500–999 grams) to prevent chronic lung disease sponsored by National Institute of Child Health & Human Development [22]. Enrollment was stopped at 360 babies because of an increase in spontaneous gastrointestinal (GI) perforation in the hydrocortisone-treated group. The primary benefit outcome for the study was survival without supplemental oxygen at 36 weeks postmenstrual age. While low-dose hydrocortisone did not significantly improve survival without supplemental oxygen in the overall study population, within the subgroup of babies exposed to chorioamnionitis (an a priori subgroup of interest), the hydrocortisone-treated group had significantly higher survival without supplemental oxygen. A benefit-risk analysis allows further examination of the relationship between survival without supplemental oxygen and GI perforation in the chorioamnionitis subgroup. Table 1 shows the proportion of babies exposed to chorioamnionitis in each treatment group that showed benefit or experienced a GI perforation.

**Table 1:** Survival without supplemental oxygen and GI perforation rates in the PROPHET study by treatment

	Placebo (n = 76)	Hydrocortisone (n = 73)
Survival without O_2_	18/76 (24%)	28/73 (38%)
GI Perforation	1/76 (1%)	8/73 (11%)
Survival without O_2 _and GI Perforation	0/76	3/73 (4%)

**Figure 3:**
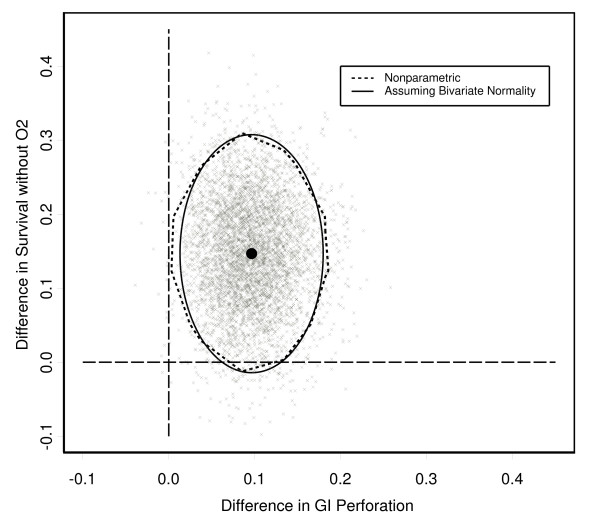
**Confidence regions and bootstrap estimates for the PROPHET study**. 90% confidence regions and bootstrap estimates for the PROPHET study.

Using Fieller's theorem, the benefit-risk ratio for the chorioamnionitis subgroup is 1.52 (90% confidence interval: 0.23 to 5.25). Thus, about 3 additional babies will survive without supplemental oxygen for every 2 GI perforations incurred from using hydrocortisone instead of placebo. We note in this case that the confidence interval is wide and is not inconsistent with as many as 5 babies benefiting for each additional adverse event incurred when hydrocortisone is used in place of placebo. The 90% confidence ellipse assuming bivariate normality and 90% nonparametric confidence region based on 5000 bootstrap samples are shown in Figure 3. The bootstrap estimates for the 5000 samples also are shown. Despite the small expected cell counts for GI perforations in the placebo and hydrocortisone groups, for this example the nonparametric and bivariate normal regions are very similar.

As a hypothetical example of choosing regions of interest for the PROPHET study, we separate the benefit-risk plane into the following regions:

**1**. Appreciable Risk: Risk difference > 0.10

**2**. Acceptable Risk: Risk difference ≤ 0.10

**a**. Hydrocortisone Superior: Benefit difference > 0.20

**b**. No Conclusion: 0.10 ≤ Benefit difference ≤ 0.20

**c**. No Appreciable Benefit: Benefit difference < 0.10

Estimates of the probabilities of falling into the selected regions are given in Table 2. The bootstrap proportions and posterior probabilities are similar and show that there is a greater than 50% chance of falling into the region of acceptable risk. However, within the acceptable risk region there is still a substantial chance that no conclusion can be reached.

**Table 2:** Estimated probabilities of falling into selected regions of interest

Region	Proportion of Bootstrap Estimates	Posterior Probability
Appreciable risk	0.44	0.46
Hydrocortisone superior	0.14	0.13
No conclusion	0.27	0.27
No appreciable benefit	0.15	0.14

Total	1	1

**Figure 4:**
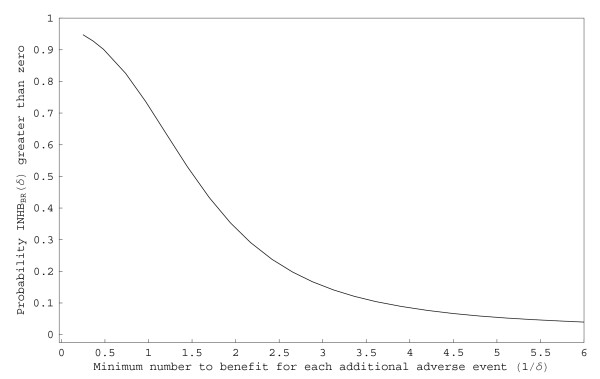
***PR*[*INHB*_*BR*_(*δ*) > 0] over a range of 1/*δ *values for the PROPHET study**. Posterior probabilities that the incremental net health benefit (*INHB*_*BR*_) of hydrocortisone compared to placebo exceeds zero as a function of the minimum number of babies who should survive without supplemental oxygen for each additional GI perforation.

Alternatively, Figure 4 shows a plot of the probability the incremental net health benefit (*INHB*_*BR*_) of hydrocortisone compared to placebo exceeds zero over a range of 1/*δ*, which can be interpreted here as the minimum number of babies who should survive without supplemental oxygen for each additional GI perforation incurred. If the threshold is one additional survivor without supplemental oxygen for each additional GI perforation, the probability *INHB*_*BR*_(1)exceeds zero is approximately 0.72. This probability quickly drops off and falls below 50% when the threshold is approximately 1.5 additional survivors without supplemental oxygen for each additional GI perforation.

These findings are not conclusive and demonstrate the need for additional study to determine how hydrocortisone therapy might be used to provide benefit in these extremely low birth weight infants without increasing risk of GI perforation. One area of potential investigation is related to indomethacin therapy's role in the development of GI perforation. There is evidence in the PROPHET study of an interaction between hydrocortisone and early indomethacin therapy, although indomethacin was not randomized in this trial. In the absence of early indomethacin, low-dose hydrocortisone therapy administered as described for this study has not previously been associated with increased incidence of GI perforation [23]. For this analysis S-PLUS was used to construct the confidence ellipse and nonparametric region. The two-dimensional kernel density estimation function *kde *and the ellipse-drawing function *ellipse *for S-PLUS or R are available from StatLib [24]. Mathematica was used to compute the benefit-risk ratio and associated confidence interval and all posterior probabilities, but these computations also can be done using S-PLUS or R.

## Conclusion

It is less ambiguous to jointly look at the difference in risk and difference in benefit in the benefit-risk plane than to collapse information by computing a benefit-risk ratio. If the benefit-risk ratio is reported, the joint distribution of benefit and risk also should be presented. When looking at the joint distribution, uncertainty in benefits and risks can be represented by confidence ellipses based on the assumption of bivariate normality or plots of estimates from bootstrap samples with or without a nonparametric confidence region. To quantify the probability of falling into regions of interest, the proportion of bootstrap estimates or posterior probabilities can be computed for particular regions. Bayesian methods provide a flexible framework in which to summarize the joint distribution of benefit and risk. Using the Bayesian framework allows one to easily conduct benefit-risk analyses similar to the incremental net health benefit analyses used for cost-effectiveness research. As this approach is based on linear combinations of benefit and risk, many of the inferential problems associated with ratios are avoided.

We have chosen to focus on the comparison of two therapies for a binary measure of benefit and a binary measure of risk, as the motivating PROPHET study had a binary primary benefit outcome and an increased rate of a single adverse event, spontaneous GI perforation, which resulted in an early stop of the trial. However, the Bayesian methods easily generalize to allow for other distributions of benefit and risk, provided one can simulate samples from the posterior distribution of interest. The Bayesian methods also allow prior information to be incorporated into the inference if such information is available. When it is of interest to compare more than two therapies, the benefit-risk approaches shown can be conducted in a pairwise fashion.

## Competing interests

The authors declare that they have no competing interests.

## Authors' contributions

MS developed and conducted the statistical analysis and drafted the manuscript. KW conceived of and led the PROPHET study which serves as the illustrative example. All authors participated in the interpretation of the analysis and read and approved the final manuscript.

## Pre-publication history

The pre-publication history for this paper can be accessed here:


